# Supplementary material for: The Prevalence and Impact of Polycystic Ovary Syndrome in Recurrent Miscarriage: A Retrospective Cohort Study and Meta-Analysis
Source: J Clin Med. 2020 Aug 21;9(9):2700. doi: 10.3390/jcm9092700 (PMC7565166; doi:10.3390/jcm9092700)
Supplement: Supplementary file 1 [file jcm-09-02700-s001.pdf]

Supplementary Material

The Prevalence and Impact of Polycystic Ovary Syndrome in Recurrent Miscarriage: A Retrospective Cohort Study and Meta-Analysis

Daniel Mayrhofer [1], Marlene Hager [1], Katharina Walch [1], Stefan Ghobrial [1], Nina Rogenhofer [2], Rodrig Marculescu [3], Rudolf Seemann [4] and Johannes Ott [1,*]

[1] Clinical Division of Gynecologic Endocrinology and Reproductive Medicine, Department of Obstetrics and Gynecology, Medical University of Vienna, Spitalgasse 23, 1090 Vienna, Austria; daniel.mayrhofer@gmail.com (D.M.), marlene.hager@meduniwien.ac.at (M.H.), katharina.walch@meduniwien.ac.at (K.W.), stefan.ghobrial@meduniwien.ac.at (S.G.)

[2] Division of Gynecological Endocrinology and Reproductive Medicine, Department of Obstetrics and Gynecology, University Hospital LMU Munich, Marchioninistraße 15, 81377 Munich, Germany; Nina.Rogenhofer@med.uni-muenchen.de

[3] Department of Laboratory Medicine, Medical University of Vienna, Spitalgasse 23, 1090 Vienna, Austria; rodrig.marculescu@meduniwien.ac.at

[4] Department of Oral and Maxillofacial Surgery, Medical University of Vienna, Spitalgasse 23, 1090 Vienna, Austria; rudolf.seemann@gmail.com

* Correspondence: johannes.ott@meduniwien.ac.at

Supplementary Table 1. Association of risk factors for RM with polycystic ovary syndrome.

Risk factor	Women with PCOS (*n*= 43)	Women without PCOS (*n*= 409)	*p*
Overt hypothyroidism	3 (7.0)	58 (14.2)	0.244
Overt hyperthyroidism	0	11 (2.7)	0.406
Antiphospholipid syndrome	1 (2.3)	16 (3.9)	0.719
Any thrombophilic defect	9 (20.9)	32 (7.8)	0.010
Any uterine abnormality	7 (16.3)	58 (14.2)	0.819
Bacterial vaginosis (including infection with ureaplasm and mycoplasma hominis)	3 (7.0)	20 (4.9)	0.713
Other RM negative for selected riskfactors	28 (65.1)	153 (37.4)	<0.001

Data are provided as number (frequency); statistical differences were tested using the Fisher*s exact test.

Supplementary Table 2. "Leave-one-out" sensitivity analysis for the meta-analysis on PCOS prevalence on RM. Estimates and 95% confidence intervals obtained in a leave-one-out sensitivity analysis of all three eligible studies. The calculations are performed as in the main analysis. However, in each round of the sensitivity analysis one study is not included.

	Estimate pooled	95% confidence interval
All studies included	0.143	0.062; 0.249
Without Cocksedge et al. [11]	0.165	0.044; 0.342
Without Matjila et al. [24]	0.097	0.077; 0.120
Without Mayrhofer et al.	0.169	0.050; 0.338

Supplementary Table 3. "Leave-one-out" sensitivity analysis for the meta-analysis on PCOM prevalence on RM. Estimates and 95% confidence intervals obtained in a leave-one-out sensitivity analysis of all four eligible studies. The calculations are performed as in the main analysis. However, in each round of the sensitivity analysis one study is not included.

	**Estimate pooled**	**95% confidence interval**
All studies included	0.508	0.296; 0.719
Without Rai et al. [14]	0.546	0.259; 0.818
Without Tulppala et al. [15]	0.532	0.243; 0.810
Without Liddell et al. [16]	0.560	0.291; 0.812
Without Sagle et al. [13]	0.405	0.385; 0.425
